# Testing a theory of strategic implementation leadership, implementation climate, and clinicians’ use of evidence-based practice: a 5-year panel analysis

**DOI:** 10.1186/s13012-020-0970-7

**Published:** 2020-02-07

**Authors:** Nathaniel J. Williams, Courtney Benjamin Wolk, Emily M. Becker-Haimes, Rinad S. Beidas

**Affiliations:** 1 School of Social Work, Boise State University, Boise, ID USA; 2 Institute for the Study of Behavioral Health and Addiction, Boise State University, Boise, ID USA; 3 School of Social Work, Boise State University, Room 711, 1910 University Drive, Boise, ID 83725 USA; 4 Department of Psychiatry, Perelman School of Medicine, University of Pennsylvania, Philadelphia, PA USA; 5 Department of Medical Ethics and Health Policy, Perelman School of Medicine, University of Pennsylvania, Philadelphia, PA USA; 6 Department of Medicine, Perelman School of Medicine, University of Pennsylvania, Philadelphia, PA USA; 7 Penn Implementation Science Center at the Leonard Davis Institute of Health Economics (PISCE@LDI), University of Pennsylvania, Philadelphia, PA USA; 8 Hall Mercer Community Mental Health Center, Pennsylvania Hospital, Philadelphia, PA USA

**Keywords:** Implementation leadership, Implementation climate, Behavioral health, Evidence-based practice, Mediation, Mechanism

## Abstract

**Background:**

Implementation theory suggests that first-level leaders, sometimes referred to as middle managers, can increase clinicians’ use of evidence-based practice (EBP) in healthcare settings by enacting specific leadership behaviors (i.e., proactive, knowledgeable, supportive, perseverant with regard to implementation) that develop an EBP implementation climate within the organization; however, longitudinal and quasi-experimental studies are needed to test this hypothesis.

**Methods:**

Using data collected at three waves over a 5-year period from a panel of 30 outpatient children’s mental health clinics employing 496 clinicians, we conducted a quasi-experimental difference-in-differences study to test whether within-organization change in implementation leadership predicted within-organization change in EBP implementation climate, and whether change in EBP implementation climate predicted within-organization change in clinicians’ use of EBP. At each wave, clinicians reported on their first-level leaders’ implementation leadership, their organization’s EBP implementation climate, and their use of both EBP and non-EBP psychotherapy techniques for childhood psychiatric disorders. Hypotheses were tested using econometric two-way fixed effects regression models at the organization level which controlled for all stable organizational characteristics, population trends in the outcomes over time, and time-varying covariates.

**Results:**

Organizations that improved from low to high levels of implementation leadership experienced significantly greater increases in their level of EBP implementation climate (*d* = .92, *p* = .017) and within-organization increases in implementation leadership accounted for 11% of the variance in improvement in EBP implementation climate beyond all other covariates. In turn, organizations that improved from low to high levels of EBP implementation climate experienced significantly greater increases in their clinicians’ average EBP use (*d* = .55, *p* = .007) and within-organization improvement in EBP implementation climate accounted for 14% of the variance in increased clinician EBP use. Mediation analyses indicated that improvement in implementation leadership had a significant indirect effect on clinicians’ EBP use via improvement in EBP implementation climate (*d* = .26, 95% CI [.02 to .59]).

**Conclusions:**

When first-level leaders increase their frequency of implementation leadership behaviors, organizational EBP implementation climate improves, which in turn contributes to increased EBP use by clinicians. Trials are needed to test strategies that target this implementation leadership–EBP implementation climate mechanism.

Contributions to the literature

- Little is known about the specific actors who are most instrumental in successful evidence-based practice (EBP) implementation in healthcare settings, or about the specific behaviors these actors can use to support implementation success. Filling this knowledge gap is crucial to developing and targeting effective implementation strategies.
- Using data collected from 30 clinics across a 5-year period and incorporating a host of rigorous statistical controls, our findings confirm for the first time that when first-level leaders increase their use of specific implementation leadership behaviors, they contribute to improvements in their organization’s EBP implementation climate, which in turn contributes to increased use of EBP by clinicians.
- These findings fill knowledge gaps regarding the specific mechanisms that support EBP implementation in healthcare settings by providing robust new evidence that first-level leaders can improve EBP implementation by using specific implementation leadership behaviors to create a supportive EBP implementation climate within their clinics.

## Background

Increasing the delivery of evidence-based practices (EBP) in community settings is a major goal of efforts to transform health and behavioral health care delivery globally given widespread evidence of research-to-practice gaps in the quality and effectiveness of health services [1–4]. While implementation science has contributed myriad frameworks, methods, and outcomes over the past two decades [5], there is an increasing call for understanding the mechanisms or levers that can be targeted to improve the adoption, implementation, and sustainment of EBPs in health and behavioral healthcare systems [6–8]. Mechanisms represent causal processes through which an outcome occurs [9–11]. To date, many implementation frameworks have proposed variables that are conceptually related to EBP implementation [12, 13], and investigators have begun to validate measures of these constructs [14] and study how these constructs relate to each other and to implementation outcomes in cross-sectional studies [15]. An essential next step is to test whether changes in these variables contribute to changes in implementation outcomes using experimental, longitudinal, and/or quasi-experimental designs that incorporate rigorous controls [6, 9]. In this study, we advance understanding of the mechanisms that influence EBP implementation by examining how changes in organizational leadership and climate influence clinicians’ use of EBP across a 5-year period in a large sample of community-based behavioral health agencies that serve youth [16].

### The role of first-level leaders in EBP implementation

Implementation frameworks posit the importance of organizational leaders and their use of specific leadership behaviors as potential mechanisms to improve EBP implementation in healthcare settings [17–20]. These frameworks converge on the importance of leaders at multiple levels—from executives who make decisions about whether to implement an EBP to first-level leaders who directly supervise clinicians [21–23]—as well as on the importance of aligning leadership across levels [24–26]. Furthermore, many frameworks suggest the unique importance of first-level leaders, also known as middle managers, who directly supervise frontline clinicians [21, 27]. First-level leaders are believed to be important for supporting EBP implementation because they have frequent interpersonal contact with clinicians, they play a prominent role in supervision and guiding clinical care, and they form a bridge between executives who often make decisions about the adoption of EBPs and clinicians who are tasked with implementing EBPs with clients [28, 29].

**Fig. 1:**
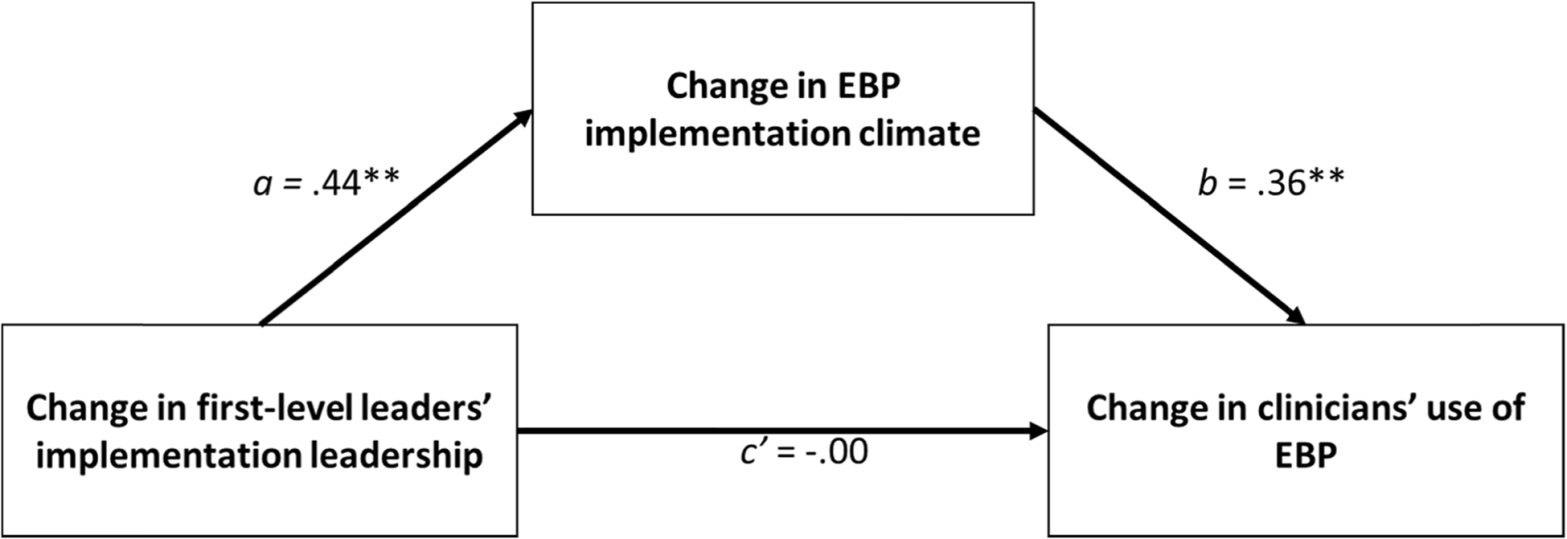
Study theoretical model. Hypothesis 1 states that within-organization increases in first-level leaders’ use of implementation leadership behavior will improve the EBP implementation climate within their organization (path *a*). Hypothesis 2 states that within-organization increases in EBP implementation climate will contribute to increases in clinicians’ use of EBP, controlling for implementation leadership (path *b*). Hypothesis 4 states that within-organization increases in implementation leadership will indirectly improve clinicians’ EBP use via within-organization improvement in EBP implementation climate (path *a* × path *b*). Path coefficients are estimated using econometric two-way fixed effects regression models at the organization level; they represent the relationships between within-organization change in the antecedent and within-organization change in the consequent, controlling for all stable organizational characteristics, population trends in the consequents over time, and time-varying covariates of molar organizational climate, transformational leadership, and workforce composition (see Table 3, *k* = 73, *N* = 30)

In this study, we test a theory of implementation leadership proposed by Aarons et al. [24, 27, 30] which posits specific hypotheses regarding the types of behaviors first-level leaders can use to influence clinicians’ EBP implementation behavior and the specific mechanism through which these behaviors influence clinicians’ practice. As is shown in Fig. 1, this theory suggests that when first-level leaders use a specific set of behaviors referred to as ‘implementation leadership,’ they will improve clinicians’ EBP implementation by creating an EBP implementation climate within the organization that conveys strong expectations, support, and rewards for the use of EBP [31–33]. In turn, the creation of an EBP implementation climate within the organization serves as the most proximal, salient, and powerful organization-level antecedent to clinicians’ EBP implementation behavior [30].

EBP implementation climate, defined as the extent to which employees share perceptions that the adoption and implementation of EBP is expected, supported, and rewarded within their organization [31, 34, 35], is hypothesized to be important for shaping clinicians’ implementation behavior in part due to the inherent difficulty in closely monitoring and controlling the processes of healthcare delivery [36]. In contrast to manufacturing and production processes, which are highly routinized, predictable, and observable, the process of delivering behavioral healthcare is non-routinized and often unpredictable, simultaneously produced by clinicians and consumed by patients, and occurs in private encounters [37]. These characteristics make it difficult and expensive to ensure EBPs are delivered with fidelity during every clinical encounter. As an alternative, organizational leaders can align organizational policies, procedures, practices, incentives, and supports to create an EBP implementation climate that shifts clinicians’ attitudes and motivation toward the effective use of EBPs in their practice [31, 33, 38]. When clinician perceptions of EBP implementation climate are high, it signals a shared belief that EBP use is a true and lasting priority for the organization rather than a passing trend that can be ignored. Cross-sectional studies demonstrate that healthcare organizations vary significantly in their levels of EBP implementation climate and that clinicians in organizations with higher levels of EBP implementation climate have better attitudes toward EBPs and use them to a greater extent than those in organizations with low levels of EBP implementation climate [39–42]. Data are lacking, however, on whether and how EBP implementation climate changes over time and how these changes relate to change in clinicians’ behavior.

First-level leaders are posited to play a key role in shaping EBP implementation climate because they are the most salient and immediate representation of the organization’s expectations, policies, procedures, and practices which form a basis for clinicians’ climate perceptions [28]. From an interactional standpoint, first-level leaders use both general transformational leadership and strategically focused implementation leadership behaviors to influence clinicians’ perceptions of EBP implementation climate [36]. As described by the full-range leadership model, transformational leadership refers to a set of general leader behaviors that are applicable across many settings where the leader wishes to inspire and motivate followers to pursue an ideal or a course of action [43]. Leaders who are transformational serve as idealized role-models of the qualities and behaviors they hope their followers will enact. They present a value-based vision that inspires action and they engage their followers’ intellect in co-resolving critical challenges within the context of supportive relationships [43]. Within Aaron et al.’s model [24, 27, 30], these general leadership behaviors form a necessary but not sufficient foundation on which the leader builds to improve EBP implementation.

In contrast to the generalized and broadly applicable transformational leadership behaviors, implementation leadership refers to strategically focused leader behaviors that reflect the leader’s commitment to, support for, and perseverance during EBP implementation [27, 36, 44]. Whereas transformational leadership provides a foundation of trust for the leader-clinician relationship, implementation leadership focuses that relationship, in part, on integrating the use of EBP in clinical practice. Drawing on the concept of climate embedding mechanisms, which represent behaviors leaders use to create a specific type of climate within their organization [24, 45], Aarons et al. [27] posit that implementation leadership is exhibited through behaviors of (a) proactively planning for and removing obstacles to implementation, (b) demonstrating and applying knowledge regarding the specific EBP being implemented, (c) supporting and appreciating followers’ efforts to implement the EBP, and (d) responding effectively to challenges and persevering with EBP implementation even when it becomes difficult [27]. These implementation-focused leadership behaviors complement general transformational leadership; together, the two types of leadership contribute to a positive leader-follower relationship that is focused on the strategic imperative of effectively integrating EBP into care delivery [30]. Cross-sectional studies have shown that higher levels of implementation leadership among first-level leaders are linked to higher levels of EBP implementation climate [40, 46, 47] and superior clinician attitudes toward EBP [22]; however, we are unaware of any longitudinal studies that examine how implementation leadership changes over time or that link these changes to changes in EBP implementation climate.

In order for implementation leadership to serve as a mechanism that influences clinicians’ EBP implementation behaviors, it must be true that implementation leadership is malleable, and that changes in implementation leadership contribute to change in clinicians’ behavior either directly or indirectly (e.g., via change in EBP implementation climate) [6, 7]. In this study, we address both of these issues by examining how, and if, implementation leadership changes naturalistically over time within behavioral healthcare organizations, and by testing whether these changes are associated with changes in an organization’s EBP implementation climate and clinicians’ EBP use. In addition, we test whether Aarons et al.’s [24, 27, 30] theory has discriminant validity by examining whether changes in implementation leadership and EBP implementation climate are related to change in clinicians’ non-EBP practice behaviors. An important premise of the theory is that implementation leadership and EBP implementation climate have specific and narrowly defined effects on EBP use. If changes in implementation leadership and EBP implementation climate predict change in other, non-EBP practice behaviors, this would call into question the validity of the theory or the validity of the measures used to test the theory.

### Study hypotheses

Hypothesis 1: Within-organization increases in implementation leadership by first-level leaders will predict within-organization increases in the level of EBP implementation climate.

Hypothesis 2: Within-organization increases in EBP implementation climate will predict within-organization increases in clinicians’ EBP use.

Hypothesis 3:Within-organization increases in EBP implementation climate will *not* predict within-organization increases in clinicians’ use of non-EBP techniques (evidence of discriminant validity).

Hypothesis 4: Within-organization increases in EBP implementation leadership will indirectly increase clinicians’ EBP use via increases in the level of EBP implementation climate (i.e., mediation).

Together, these hypotheses (a) test the implementation leadership–EBP implementation climate mechanism described above and shown in Fig. 1, and (b) provide discriminant validity evidence for the model by testing whether hypothesized changes in self-reported technique use were specific to EBP or occurred for both EBP and non-EBP techniques.

## Methods

### Study setting and design

Using data collected at three waves over a 5-year period from a panel of 30 outpatient children’s mental health clinics [48], we conducted a quasi-experimental difference-in-differences study [49, 50], incorporating econometric two-way fixed effects regression models [51], to test whether within-organization changes in implementation leadership were associated with changes in EBP implementation climate and ultimately with changes in clinicians’ EBP use. Difference-in-difference designs that use data from a longitudinal panel are the gold standard quasi-experimental approach because they permit investigators to isolate the relationships between within-unit change in presumed antecedents and consequents by eliminating all stable, between-unit confounds that may explain variation in the outcome of interest and by controlling for population trends in the consequents over time [49].

In a classic difference-in-differences design, longitudinal data are collected on a set of units that never experience the exposure (i.e., an organization that has continually low implementation leadership/ EBP implementation climate) and on a set of units that experience the exposure after a baseline period of time (i.e., an organization where implementation leadership/ EBP implementation climate shifts from low to high). Under the common trend assumption, the effect of the exposure is estimated as the difference between the change in the outcome that occurs in the exposed group less the change that occurs in the unexposed group (i.e., difference-in-differences) [49]. This approach can be generalized to studies in which units experience the exposure at different times (i.e., changes in implementation leadership/ EBP implementation climate occur at different waves), and move in and out of a state of exposure, through the use of two-way fixed effects regression models [49]. In addition, two-way fixed effects regression models can be used to estimate relationships between within-unit change in continuous antecedent and consequent variables [51]; in these analyses, each unit (i.e., each organization) serves as its own control and the effects that are estimated between continuous variables represent within-unit effects [51]. In this study, we applied these approaches to examine the relationships between organization-level changes in antecedents and consequents within 30 outpatient children’s mental health clinics measured at three waves across a 5-year period. We followed the STROBE checklist for reporting elements of this longitudinal, quasi-experimental study [52] (see Additional file 1).

The organizations in this study were embedded within a publicly funded behavioral health system and full details of the larger study, including its overall design and the system-level context within which it occurred are published elsewhere [16, 53]. In brief, beginning in 2013, the City of Philadelphia in the USA launched the Evidence Based Practice and Innovation Center (EPIC) to improve EBP implementation in its Medicaid-funded behavioral health provider network which serves over 600,000 persons annually [54]. EPIC supports EBP implementation on a system-wide basis through a coordinated set of policy, fiscal, and technical assistance activities which are described elsewhere [55]. During the study period, EPIC supported the implementation of cognitive behavioral psychotherapies with strong empirical support for treating a range of psychiatric disorders, including cognitive therapy, [56], prolonged exposure, [57], trauma-focused cognitive-behavioral therapy [58], dialectical behavior therapy [59], and parent-child interaction therapy [60].

Recognizing the opportunity this system-level effort presented, our research team partnered with the City of Philadelphia to assess changes in clinicians’ use of EBP during 5 years and to examine how these changes related to changes in organizational characteristics presumed to influence EBP implementation [48]. Whereas previous work evaluated the effectiveness of EPIC [48], the focus of this study was to test the theoretical model shown in Fig. 1. The 5-year study period began immediately prior to the launch of EPIC in 2013 and continued through 2017. The present study uses data from all three waves including assessments completed at baseline (2013), 2-year follow-up (2015), and 5-year follow-up (2017).

### Participants

The study incorporated two levels of sampling—organizations and clinicians. At the organization level, beginning in 2012, we purposively sampled publically funded organizations that deliver outpatient mental health services to Medicaid-eligible youth in Philadelphia. Given that Philadelphia has a single payer system (Community Behavioral Health; CBH) for public behavioral health services, we obtained a list from the payer of all organizations that had submitted a claim in 2011–2012. There were over 100 organizations delivering outpatient services to youth. Our intention was to use purposive sampling to generate a representative sample of the organizations that served the largest number of youth in the system. We selected the first 29 organizations as our population of interest because together they serve approximately 80% of youth receiving publically funded behavioral health care. The majority of the remaining organizations in the system were very small and did not employ many clinicians and/or see many youth. The organizations that we recruited were geographically spread across Philadelphia county and ranged in size with regard to number of youth served. Over the course of the study, we enrolled 22 of the 29 organizations (76%). Some organizations had multiple sites with distinct leadership structures and operations at each site; these were treated as separate units in the analysis for a total of *N* = 31 sites, which we refer to as organizations in what follows. Of the 31 organizations that participated in the study across the 5-year study period, 18 participated in three waves, nine participated in two waves, and four participated in one wave. One organization was a multivariate outlier on study measures at wave 3, resulting in a final analytic sample of *N* = 30 organizations with a total of *k* = 73 organization-level observations across three waves (average of 2.4 observations per organization).

The second level of sampling included all clinicians who worked with youth within each organization at each wave. Specifically, with the permission of organizational leaders, researchers scheduled group meetings with all clinicians working within the organizations that delivered youth outpatient services, during which the research team presented the study, obtained written informed consent, and collected measures onsite. The only inclusion criterion was that clinicians deliver behavioral health services to youth (clients under age 18) via the outpatient program. We did not exclude any clinicians meeting this criterion and included clinicians-in-training (e.g., interns).

In total, 496 clinicians were included in the analytic sample of 30 organizations. Within this group, 387 clinicians (78%) provided data once; 94 clinicians (19%) provided data twice, and 15 clinicians (3%) provided data three times. The majority of clinicians were female (81%) and came from diverse racial and ethnic backgrounds (47% white, 27% African American, 15% ‘Other Race,’ 6% Asian, < 1% Native Hawaiian or Other Pacific Islander or American Indian or Alaska Native, 19% Latino). The average age was 37.3 years (SD = 11.41) with an average of 8.6 years (SD = 8.5) of experience in human services and 2.7 years (SD = 3.9) in their current agency. Ten percent of clinicians had a doctoral degree and the remainder had master’s or bachelor’s degrees. Clinicians’ demographic characteristics did not vary by wave (all *p*’s > .10). The two-level sampling approach allowed us to examine changes in implementation leadership, EBP implementation climate, and EBP use at the organization level over time without assuming that individual clinicians were the same at each wave.

### Procedures

Clinicians completed study questionnaires in 2013, 2015, and 2017 during a 1- to 1.5-h meeting with researchers at their organization during regular work hours. In order to assure confidentiality and minimize demand characteristics, organizational leaders were not present. Questionnaires addressed clinicians’ use of psychotherapy techniques with a representative client, first-level leader implementation behavior, clinician perceptions of their organization’s EBP implementation climate and general organizational climate, as well as clinician professional and demographic characteristics. Questionnaires were returned directly to researchers and clinicians were assured that organizational leaders would not have access to their individual-level data. Participants received $50 USD for their time. All study procedures were approved by the City of Philadelphia Institutional Review Board and the University of Pennsylvania Institutional Review Board.

### Measures

#### Dependent variables

Clinicians’ self-reported *use of EBP* was measured using the 33-item cognitive-behavioral therapy (CBT) subscale of the Therapy Procedures Checklist-Family Revised (TPC-FR) [61]. Scores on this scale indicate the frequency with which clinicians use cognitive-behavioral techniques with a representative client. Prior research supports the TPC-FR’s test-retest reliability, criterion-related validity, and within-therapist sensitivity to change [61, 62]. The measure presents a list of specific psychotherapy techniques derived from three well-established models (CBT, family therapy, and psychodynamic therapy) and clinicians indicate the extent to which they use each technique with a current client who is representative of their larger caseload. Item responses are made on a continuum ranging from 1 (*rarely*) to 5 (*most of the time*). We used the CBT subscale as the primary criterion variable in this study because of the strong empirical support for the effectiveness of CBT in the treatment of youth psychiatric disorders [63–67] and because the larger system initiative supported this model. Coefficient alpha for this scale was α = .93 in the sample.

In order to provide discriminant validity evidence for our theoretical model, we also used the 16-item psychodynamic subscale of the TPC-FR as an outcome indicative of clinicians’ *use of non-EBP* techniques for hypothesis 3. Psychodynamic techniques have weaker empirical support for the treatment of psychiatric disorders among youth [68, 69]. Coefficient alpha was α = .85 in the sample.

In order to align our levels of theory and analysis, we aggregated (i.e., averaged) clinicians’ responses on the CBT and psychodynamic subscales to the organization level at each wave. Thus, our dependent variables represented the average frequency with which clinicians used cognitive-behavioral (i.e., EBP) or psychodynamic techniques (i.e., non-EBP) with a representative client at each wave [70, 71].

#### Independent variables

Clinicians rated the *implementation leadership* of their first-level leader (i.e., their direct supervisor) using the 12-item Implementation Leadership Scale (ILS) [27]. This scale assesses a leader’s behavior with regard to (a) proactiveness in promoting EBP implementation, (b) knowledge of EBP and using this to support clinicians, (c) daily support of EBP implementation, and (d) perseverance through the ups and downs of EBP implementation. Responses are made on a 0 (*not at all*) to 4 (*very great extent*) scale and a total score is derived by averaging the items. Psychometric studies indicate scores on the ILS exhibit excellent internal consistency and convergent and discriminant validity [27]. Coefficient alpha in the sample was α = .98.

Clinicians’ perceptions of their organization’s *EBP implementation climate* were measured using the total score of the 18-item Implementation Climate Scale (ICS) [31]. This scale addresses six subdomains which capture the extent to which an organization focuses on EBP, provides educational support for EBP, recognizes clinicians for excellence in EBP, rewards clinicians for demonstrating expertise in EBP, selects clinicians based on their EBP acumen, and selects clinicians for general openness. Responses are made on a 0 (*Not at All*) to 4 (*A Very Great Extent*) scale and a total score is calculated as the mean across all items. Prior research supports the ICS’s structural validity, total score reliability, and convergent and discriminant validity [31, 41, 46]. Coefficient alpha for this scale was α = .94 in the sample.

#### Control variables

Two-way fixed effects regression models control for all stable organizational characteristics and for population trends in the outcome over time; however, they do not control for potential time-varying confounds within organizations from wave-to-wave (e.g., wave-to-wave change in general leadership or climate) [51]. To address this, we included the following variables as covariates in all analyses.

*Molar organizational climate* is defined as clinicians’ shared perceptions of the overall impact of the work environment on their personal well-being; that is, whether the work environment is a ‘good’ or ‘bad’ place to work [72]. We assessed it using the well-established, 15-item *functionality* scale of the Organizational Social Context (OSC) measure to account for the general work environment and because prior research indicates that this characteristic is related to clinicians’ use of EBP [53, 73, 74]. Prior research supports this scale’s structural and criterion-related validity [73, 75, 76]. Responses are made on a five-point scale from 1 (*Never*) to 5 (*Always*). Coefficient alpha was α = .92 in the sample.

First-level leaders’ *transformational leadership* was included in the models in order to isolate the effects of implementation leadership (vs. transformational leadership) on EBP implementation climate and because prior research has linked transformational leadership to EBP implementation climate, clinicians’ attitudes toward EBP, and EBP adoption and sustainment [42, 77–80]. Clinicians rated the extent to which their first-level leaders exhibited transformational leadership behaviors using the Multifactor Leadership Questionnaire [81, 82], which is one of the most widely used measures of transformational leadership and has excellent psychometric properties [83]. Consistent with prior research, we used the transformational leadership total score (averaged on a 5-point scale from *Not at all* to *Frequently*, *if not always*) which incorporates four subscales: Idealized Influence, Inspirational Motivation, Intellectual Stimulation, and Individual Consideration (20 items, α = .97).

We included a single, time-varying, workforce variable—*clinicians*’ *average years of experience*—in our models based on preliminary bivariate analyses which showed that this was the only time-varying workforce characteristic associated with change in clinicians’ EBP use in our sample (*B* = .02, *SE* = .01, *p* = .031). No other time-varying workforce characteristics, including clinicians’ average attitudes toward EBP as measured using the Evidence-Based Practice Attitudes Scale [84], were associated with EBP use (all *p*’s > .10) and there was no evidence that first-level leader turnover predicted EBP use (*p* = .322). Clinicians reported on their years of experience working as a clinician and this variable was averaged to the organization-level at each wave to represent the average experience of the organization’s workforce at each wave.

#### Data aggregation

Consistent with best practices [6, 70, 85], we generated organization-level values for implementation leadership, EBP implementation climate, molar organizational climate, and transformational leadership by aggregating (i.e., averaging) clinicians’ individual responses to the organization level on these respective scales. The validity of these compositional variables was supported by high levels of inter-rater agreement within each organization, as measured using the *a*_wg(j)_ statistic; all *a*_wg(j)_ values were above the recommended cutoff of .7 in our sample [86, 87].

### Data analysis

In order to take full advantage of the data structure, we conducted two complementary sets of analyses that tested hypotheses 1–4. Both sets of analyses used econometric two-way fixed effects regression models [51], also referred to as panel linear models [88], at the organization level. First, following Wing et al. [49] and reflecting a traditional generalized difference-in-differences design, we categorized the implementation leadership and EBP implementation climate within each organization at each wave as either *high* or *low* based on a median split and used these dichotomous indicators as measures of exposure. The two-way fixed effects regression models were specified as:

$$ {Y}_{it}={\beta}_0+{\beta}_1{O}_{\mathrm{i}}+{\beta}_2{T}_{\mathrm{t}}+{\beta}_3{\mathrm{Z}}_{\mathrm{i}\mathrm{t}}+{\beta}_4{X}_{it}+{\varepsilon}_{it} $$

where *Y*_*it*_ represents the outcome for organization *i* at time *t*, *O*_*i*_ represents a set of dummy variables that control for the combined effects of all stable organizational characteristics, *T*_*t*_ represents a set of dummy variables that capture the combined effects of population trends or shocks that affect all organizations at time *t*, *Z*_*it*_ represents a vector of time-varying covariates, and *X*_*it*_ represents the dichotomous exposure variable indicating whether the organization experienced high or low implementation leadership or EBP implementation climate at time *t*. The *β*_*4*_ coefficient captures the exposure effect, that is, the mean improvement in the outcome attributable to an organization shifting from a low to a high level of either implementation leadership (in model 1) or EBP implementation climate (in model 2). Effect sizes for these models are expressed as Cohen’s *d* [89] which captures the conditional, standardized mean difference in change between organizations that shifted from low to high levels of implementation leadership (or EBP implementation climate) versus those that did not.

Second, we ran the same models but included implementation leadership and EBP implementation climate as continuous variables (rather than dichotomous indicators) in the analyses. The beta coefficients from these two-way fixed effects regression models represent the effect of within-organization change in each predictor on within-organization change in each outcome, controlling for all stable between-organization differences, population trends in the outcome over time, and the other time-varying predictors in the model. Effect sizes for these models are expressed as an incremental R-squared; that is, the percentage of variance in within-organization change in the outcome accounted for by the focal predictor over-and-above all other predictors in the model. All analyses were implemented in R using the plm package [88]. Following best practices, missing waves of data were handled using maximum likelihood estimation [90]. Following estimation of all models, we examined residual plots and other diagnostics to confirm the tenability of model assumptions.

We used the product of coefficients approach, in conjunction with the joint significance test [91, 92] and asymmetric 95% Monte Carlo confidence intervals, to test our mediation hypothesis (hypothesis 4) [93, 94]. Under this approach, regression analyses are used to estimate the two paths shown in Fig. 1 that link the independent variable to the mediator (‘path a’) and the mediator to the dependent variable, controlling for the independent variable (‘path b’). The product of these path estimates (i.e., a*b) quantifies the indirect or mediation effect [95]. The statistical significance of the mediation effect is tested using (1) the joint significance test, which represents a null hypothesis significance testing approach, and (2) asymmetric 95% confidence intervals developed via Monte Carlo simulation methods with 100,000 replications [93, 96].

## Results

### Preliminary analyses

**Fig. 2:**
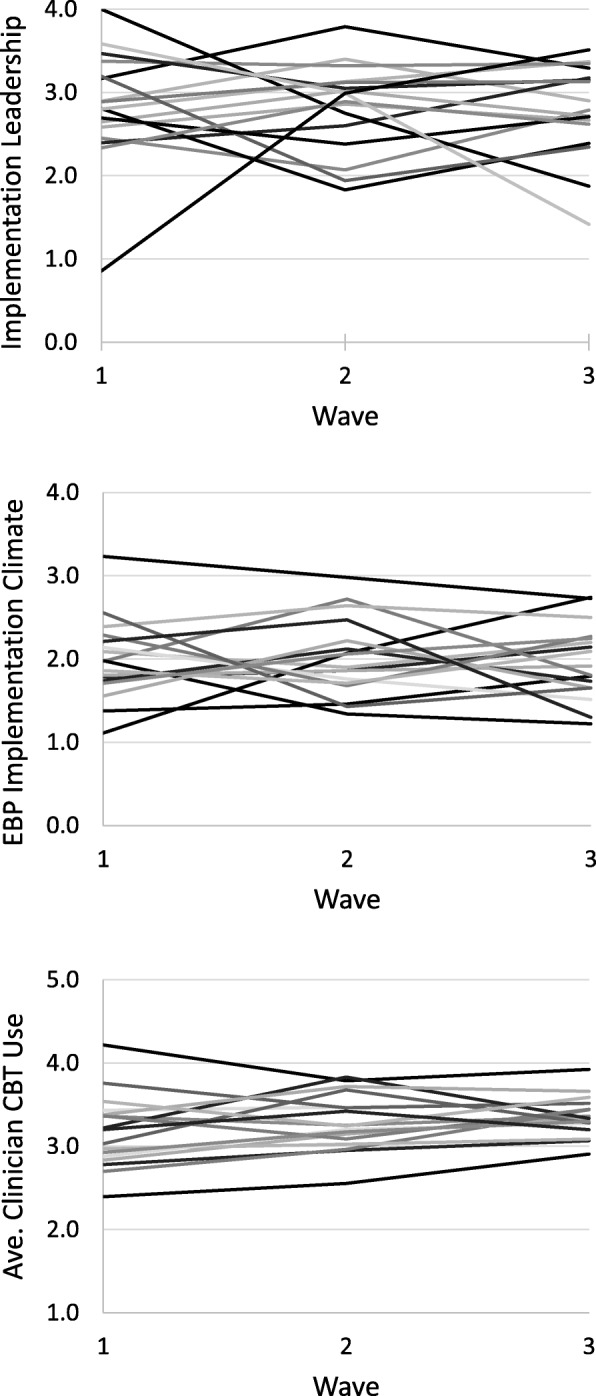
Wave-to-wave, within-organization change in implementation leadership, EBP implementation climate, and clinician CBT use. Each line depicts change in the raw observed scores of a single organization (*k* = 30). Waves are spaced approximately two years apart. *CBT* cognitive behavioral therapy, *EBP* evidence-based practice

Table 1 presents descriptive statistics for all study variables at wave 1 as well as the average within-organization change and variation in within-organization change from wave to wave. Figure 2 shows how the primary antecedent and consequent variables changed within organizations over time. In order to test whether there was significant variation across organizations in how EBP use, implementation leadership, and EBP implementation climate changed from wave to wave, we conducted a series of one-sample *t* tests. These tests compared the mean absolute value of within-organization change from wave 1 to wave 2 (and wave 2 to wave 3) to a population value of zero. All *t* tests were statistically significant (all *p*’s < .001), confirming that the absolute value of within-organization change from wave to wave was significantly different from zero for our primary antecedent and consequent variables. Furthermore, the Cohen’s *d* effect size for these (absolute value) within-organization changes were *d* = .60 for EBP use, *d* = .75 for implementation leadership, and *d* = .74 for implementation climate, representing medium effects [89]. Table 1 also shows the percentage of organizations that experienced a moderate change (defined as a > .5 standard deviation change in either direction) from wave to wave on each variable. These analyses confirm that there were statistically significant and substantively meaningful within-organization changes in the primary antecedent and consequent variables of interest during the study period.

**Table 1:** Descriptive statistics for study variables at baseline and change in variables across waves

Variable	Wave 1				Δ from wave 1 to wave 2					Δ from wave 2 to wave 3				
	*Mean*	*SD*	*Min.*	*Max.*	*Mean* Δ	*SD*	*Min.*	*Max.*	% *Δ by +/−* 0.5 *SD*	*Mean* Δ	*SD*	*Min.*	*Max.*	% *Δ by* +/− 0.5 *SD*
Ave. clinician use of CBT techniques (1–5)	3.18	.42	2.39	4.22	.11	.30	− .43	.65	63%	− .01	.33	− 1.00	.48	46%
Ave. clinician use of psychodynamic techniques (1–5)	3.35	.32	2.80	4.07	.02	.26	− .32	.76	53%	.09	.29	− .60	.73	68%
Implementation leadership (0–4)	2.79	.69	.85	4.00	− .04	.79	− 1.25	2.14	63%	.04	.58	− 1.57	1.01	54%
EBP implementation climate (0–4)	2.05	.52	1.11	3.30	.01	.54	− 1.13	.96	58%	− .01	.45	− 1.17	.67	50%
Molar organizational climate (μ = 50, σ = 10)	59.02	14.52	15.41	84.43	4.76	14.97	− 18.47	43.36	53%	− 2.43	9.04	− 22.23	11.81	47%
Transformational leadership (0–4)	2.76	.67	.74	3.58	.05	.74	− 1.42	2.21	42%	.13	.56	− 1.12	1.29	58%

*K* = 73 observations across *N* = 30 organizations. *CBT* cognitive behavioral therapy; Δ = change; % Δ by +/− 0.5 *SD* = percent of organizations that changed by plus or minus one-half a standard deviation which is equal to a moderate effect size (Cohen’s *d*). Waves are spaced approximately 2 years apart

### Effect of implementation leadership on EBP implementation climate

Table 2 presents the difference-in-differences analyses testing hypotheses 1–3; Table 3 presents similar analyses but includes implementation leadership and EBP implementation climate as continuous variables.

**Table 2:** Generalized difference-in-differences analyses testing study hypotheses

	Consequents								
	EBP implementation climate			Clinician use of EBP			Clinician use of non-EBP		
Exposures and controls	*B*	*SE*	*p*	*B*	*SE*	*p*	*B*	*SE*	*p*
High EBP implementation climate				.23	.08	.007	.08	.08	.311
High implementation leadership	.48	.19	.017	− .03	.10	.740	− .06	.10	.545
Transformational leadership	− .08	.21	.697	− .08	.10	.425	− .18	.10	.095
Molar organizational climate	.02	.01	.087	− .01	.01	.335	.01	.01	.248
Clinicians’ average years of experience	− .02	.02	.343	.03	.01	.007	.02	.01	.037
Cohen’s *d*	.92			.55			.25		

*K* = 73 observations across *N* = 30 organizations. These are two-way fixed effects regression models. Exposures for implementation leadership and EBP implementation climate are coded as Low = 0 and High = 1 based on a median split. *EBP* evidence-based practice. EBP use is measured as clinicians’ use of cognitive-behavioral psychotherapy techniques; non-EBP use is measured as clinicians’ use of psychodynamic psychotherapy techniques. The indirect effect of exposure to improved implementation leadership on clinicians’ EBP use via improved EBP implementation climate is *d* = .26

**Table 3:** Continuous variable analyses testing study hypotheses

	Consequents								
	EBP implementation climate			Clinician use of EBP			Clinician use of non-EBP		
Antecedents	*B*	*SE*	*p*	*B*	*SE*	*p*	*B*	*SE*	*p*
EBP implementation climate				.36	.13	.009	.10	.13	.424
Implementation leadership	.44	.14	.004	− .00	.12	.976	− .13	.12	.305
Transformational leadership	− .10	.16	.546	− .15	.13	.245	− .12	.13	.359
Molar organizational climate	.01	.01	.101	− .01	.01	.239	.01	.01	.179
Clinicians’ average years of experience	− .02	.01	.197	.03	.01	.011	.02	.01	.030
Model *R*^2^	.59			.33			.19		

*K* = 73 observations across *N* = 30 organizations. These are two-way fixed effects regression models which estimate the conditional, within-organization effect of change in each antecedent variable on change in the consequent, controlling for all other variables in the model as well as population trends in the consequent over time and all stable organizational characteristics. *EBP* evidence-based practice. EBP use is measured as clinicians’ use of cognitive-behavioral psychotherapy techniques; non-EBP use is measured as clinicians’ use of psychodynamic psychotherapy techniques. Indirect effect of implementation leadership on clinician EBP use via EBP implementation climate = .16 (95% CI = .03 to .33)

Hypothesis 1 was supported by both sets of analyses. As is shown in Table 2, organizations that improved from low to high levels of implementation leadership experienced significantly greater increases in their level of EBP implementation climate compared to organizations that did not change in their level of implementation leadership (*B* = .48, *SE* = .19, *p* = .017). This represents a large effect of *d* = .92 according to criteria suggested by Cohen [89].

As is shown in Table 3, similar results were obtained in the analyses that included implementation leadership and EBP implementation climate as continuous variables: within-organization increases in implementation leadership predicted within-organization improvement in the level of EBP implementation climate (*B* = .44, *SE* = .14, *p* = .004), accounting for 11% of the variance beyond all other covariates (i.e., *R*^2^_incremental_ = .11). This represents a moderate effect size [89].

### Effect of EBP implementation climate on clinicians’ EBP use

Hypothesis 2 was also supported by both sets of analyses (see Tables 2 and 3). As is shown in Table 2, organizations that improved from low to high levels of EBP implementation climate experienced significantly greater increases in their clinicians’ average use of EBP compared to organizations that experienced no change in EBP implementation climate (*B* = .23, *SE* = .08, *p* = .007), controlling for implementation leadership and all other covariates. This represented a medium effect size of *d* = .55.

Table 3 shows that similar results were obtained in the analyses that included implementation leadership and EBP implementation climate as continuous variables: within-organization improvement in EBP implementation climate predicted within-organization increases in clinicians’ use of EBP (*B* = .36, *SE* = .13, *p* = .009), controlling for change in implementation leadership and all other covariates. This represented a moderate effect size of *R*^2^_incremental_ = .14 [89]. Results of these analyses also indicated that increases in clinicians’ average years of experience predicted increased EBP use (*B* = .03, *SE* = .01, *p* = .011).

### Effect of EBP implementation climate on clinicians’ use of non-EBP

Hypothesis 3 was designed to provide discriminant validity evidence for the theorized model and it was supported by both sets of analyses (see Tables 2 and 3). Focusing on the continuous variable analyses in Table 3, within-organization change in EBP implementation climate was not related to within-organization change in clinicians’ use of non-EBP (*B* = .10, *SE* = .13, *p* = .424). The only significant predictor in this model was clinicians’ average years of experience (*B* = .02, *SE* = .01, *p* = .030).

### Indirect effect of implementation leadership on clinicians’ EBP use via EBP implementation climate

Hypothesis 4 tested the full mediation model and it was supported by the results of the joint significance test and by the asymmetric 95% confidence intervals in both sets of analyses. As described above, organizations that improved from low to high levels of implementation leadership experienced greater increases in their level of EBP implementation climate compared to organizations that did not improve in implementation leadership (see Table 2, model 1: *B* = .48, *SE* = .19, *p* = .017); and, organizations that improved from low to high levels of EBP implementation climate experienced greater increases in clinicians’ use of EBP compared to organizations that did not improve in EBP implementation climate, while controlling for implementation leadership (see Table 2, model 2: *B* = .23, *SE* = .08, *p* = .007). Based on these analyses, we reject the null hypothesis of the joint significance test and conclude that there is positive evidence for the indirect effect of implementation leadership on clinicians’ EBP use via improvement in EBP implementation climate. Furthermore, the Monte Carlo 95% confidence interval for this indirect effect did not include zero (a*b = .11, 95% CI = .01 to .25), providing additional evidence that increases in implementation leadership had a significant indirect effect on increases in clinicians’ EBP use via improvement in EBP implementation climate. Similar results were found for the analyses that included implementation leadership and EBP implementation climate as continuous variables (see Table 3; a*b = .16, 95% CI = .03 to .33).

## Discussion

This study advances implementation theory and practice by providing the first mechanistic test of the hypothesis that first-level leaders can improve EBP implementation in healthcare settings through the use of specific implementation leadership behaviors that create an EBP implementation climate within their organization. The study validates the theory of first-level implementation leadership proposed by Aarons and colleagues [24] by supporting the hypothesis that increased frequency of implementation leadership behaviors by first-level leaders contributes to moderate to large improvements in EBP implementation climate and that, in turn, improved EBP implementation climate contributes to moderate increases in self-reported EBP use by clinicians. Further, the study provides evidence of discriminant validity, such that these relationships were specific to the targeted implementation behaviors and did not apply to non-targeted, non-EBP behaviors. That these hypotheses were tested within a longitudinal, difference-in-differences design incorporating 30 organizations measured across a 5-year period allows us to make the strongest possible inferences about the relationships between these variables short of manipulating them in a randomized experiment [49, 51]. To our knowledge, this study is among the first to use a rigorous methodological approach [49] to support a mechanistic understanding of the relationship between constructs from leading implementation science frameworks [17, 18] and theory [19, 25, 26, 28]. As such, it represents a critical step forward in advancing recommendations for rigorous work testing mechanisms and causal theory in implementation science [7, 8, 97].

Our findings confirm the hypothesis, based on theory and previous cross-sectional research, that first-level leaders play a critical role in EBP implementation and should be a target of implementation strategies [24, 26–28]. The importance of first-level leaders for creating an EBP implementation climate is likely due to their frequent interpersonal contact with frontline clinicians, their role as supervisors, and their status as a bridge between executives, who make decisions about specific EBP adoption, and clinicians who are tasked with implementing EBPs with clients [19, 25, 26]. Consistent with Aarons and colleagues’ theory [30, 98], our results indicate that first-level leaders bridge the gap in part by creating an EBP implementation climate that conveys high expectations, support, and rewards for the effective use of EBPs in clinical encounters. This study provides an actionable and important advance in our understanding of how to improve EBP implementation in healthcare settings by identifying specific implementation leadership behaviors that contribute to the formation of an EBP implementation climate and improved EBP use.

The implementation leadership behaviors described by Aarons et al. [27] (i.e., proactive, knowledgeable, supportive, and perseverant) have similarities to other implementation leadership theories, such as the theory of middle managers proposed by Birken et al. [21] and the concept of clinical champions [99], and further work is needed to clarify where these theories converge and where they make unique contributions. For example, both Birken et al. and Aarons et al. posit that first-level leaders influence EBP implementation by enacting behaviors that contribute to a positive EBP implementation climate within the organization. They differ, however, in framing these behaviors as climate embedding mechanisms (i.e., Aarons et al. [27]) versus commitment to the innovation and bridging communication gaps (Birken et al. [21]). Future research should clarify the extent to which the constructs from these theories represent complementary and potentially overlapping manifestations of the same underlying set of constructs versus unique approaches to conceptualizing implementation leadership. Research is also needed to develop validated measures of the constructs from Birken et al.’s theory [29].

Results of this study indicate there are important within-organization changes in implementation leadership, EBP implementation climate, and clinicians’ use of EBP across time even though the mean of these changes is sometimes close to zero because some organizations change in a positive direction and others in a negative direction. That some organizations exhibited improvement in implementation leadership and climate while others exhibited deterioration or no change, despite their shared exposure to the same outer context, underscores the mutability of inner context factors and their potential role in shaping EBP implementation [11, 12]. The implications of this for practice are that leaders can change their organizational contexts through the use of specific behaviors and that these changes can make a difference in clinicians’ behavior. From a research standpoint, these findings imply that implementation leadership and EBP implementation climate are mutable targets that could serve as the focus for implementation strategies designed to improve EBP implementation.

The next step in this line of research includes confirmative comparative effectiveness trials to test implementation strategies—such as the Leadership and Organizational Change for Implementation strategy (LOCI) [98] or the Training in Implementation Practice Leadership (TRIPLE) [100] strategy—that target the implementation leadership–EBP implementation climate mechanism identified in this study. The LOCI intervention [30, 98], which targets implementation leadership and EBP implementation climate through training and coaching for first-level leaders along with consultation with executives, has shown promise in pilot research and is currently under study in a number of settings [30, 101, 102]. The TRIPLE intervention aims to build leader skill in assessing quality of current service delivery; identifying appropriate and feasible EBPs; developing support for EBP use; assessing and increasing stakeholder engagement in improving practice; and identifying and using data to monitor quality and lead practice change. An initial pre-post evaluation of TRIPLE with first-level leaders found the program led to improvements in implementation leadership and EBP implementation climate [100]. If these strategies are found to be effective across settings, they could present a meaningful approach to influencing implementation change. However, there are a number of questions that remain to be answered, specifically with regard to dismantling these approaches [102] to better understand the relative contribution of the focus on leadership and EBP implementation climate versus individual clinician attitudinal and motivation change, as well as questions of cost-effectiveness.

Study strengths include the use of a rigorous quasi-experimental design, the development of discriminant validity evidence with regard to the outcome (i.e., EBP vs. non-EBP techniques), and specification and testing of a robust theoretical model within a large longitudinal sample. However, several limitations should be noted. First, the study utilized self-reported use of EBP which does not always correlate strongly with actual behavior [103]; future studies that utilize observational metrics of fidelity would increase rigor. Second, while we had a relatively large and diverse sample of 30 organizations incorporating 496 clinicians, this work was conducted within a single system that was actively supporting EBP implementation among providers and thus may not generalize to other systems. Useful next steps include the replication of this study in different healthcare systems in order to test the generalizability of the results and the generalizability of the theory. Third, this study was observational in nature; because we did not experimentally manipulate variables, we cannot make causal inferences. Fourth, common method variance cannot be ruled out as an explanation for our study findings, although we included several rigorous controls and provided discriminant validity evidence with regard to the outcome. Finally, these results are likely most reflective of larger organizations rather than single-clinician providers of therapy services. Further, smaller organizations may play a vital role in culturally specific or niche service providers.

## Conclusions

This study advances a mechanistic understanding of the relationships between implementation leadership, EBP implementation climate, and self-reported clinician EBP use, thus offering important targets for improving EBP implementation in clinical practice settings.

## Supplementary information

**Additional file 1.** STROBE checklist.

## Data Availability

RB and NW had full access to all of the data in the study and take responsibility for the integrity of the data and the accuracy of the data analysis. Requests for access to deidentified data can be sent to the Penn ALACRITY Data Sharing Committee by contacting Senior Research coordinator, Ms. Kelly Zentgraf at zentgraf@upenn.edu, 3535 Market Street, 3rd Floor, Philadelphia, PA 19107, 215-746-6038.

## Abbreviations

CBT: Cognitive behavioral therapy
CI: Confidence interval
EBP: Evidence-based practice
EPIC: Evidence-Based Practice and Innovation Center
ICS: Implementation Climate Scale
ILS: Implementation Leadership Scale
LOCI: Leadership and organizational change
OSC: Organizational social context
SD: Standard deviation
TPC-FR: Therapy Procedures Checklist-Family Revised
TRIPLE: Training in Implementation Practice Leadership
